# Trends in HPV vaccination initiation and completion among U.S. adolescents aged 13–17 years, 2021–2023: Findings from the National Immunization Survey–Teen

**DOI:** 10.1371/journal.pone.0349299

**Published:** 2026-06-08

**Authors:** Taylor Mullin, Jesse Plascak, Alai Tan, Jodi Ford, Henry Duah, Diane Von Ah

**Affiliations:** 1 College of Nursing, The Ohio State University, Columbus, Ohio, United States of America; 2 College of Medicine, The Ohio State University, Columbus, Ohio, United States of America; 3 The Ohio State University, Comprehensive Cancer Center, Columbus, Ohio, United States of America; 4 Marquette University, College of Nursing, Milwaukee, Wisconsin, United States of America; Federal University Otuoke, NIGERIA

## Abstract

Human papillomavirus (HPV) vaccination is a key strategy for preventing HPV-related cancers, yet series completion among U.S. adolescents remains below national public health goals. This study analyzed nationally representative data from the 2021–2023 National Immunization Survey–Teen (NIS-Teen) to describe recent patterns in HPV vaccination initiation and completion among adolescents aged 13–17 years. Complex survey–weighted analyses estimated the proportion of adolescents who had not initiated HPV vaccination, had initiated but not completed the series, or had completed the series based on provider-verified vaccination records and Advisory Committee on Immunization Practices (ACIP) age-specific dose recommendations. The analytic sample included 126,226 adolescents. Weighted estimates indicated that 39.8% were aged 13–14 years and 60.2% were aged 15–17 years; 51.2% were male and 48.8% female. HPV vaccine series completion remained stable across survey years, with completion estimates of 62.4% in 2021, 63.9% in 2022, and 62.5% in 2023. Initiation without completion ranged from 14.4% to 15.2%, while non-initiation ranged from 21.7% to 22.9%. Across all years, completion was consistently higher among older adolescents and females compared with younger adolescents and males. Despite modest gains in initiation, HPV vaccination coverage remained relatively stable during the study period. This stability, observed during the COVID-19 recovery period, may reflect preservation of uptake rather than true stagnation. These findings highlight the need for strategies emphasizing early vaccination, strong provider recommendations, and reminder-based follow-up to improve series completion and advance national immunization targets.

## Introduction

Human papillomavirus (HPV) is the most prevalent sexually transmitted infection globally and a major etiologic agent for multiple cancers, including cervical, anal, vulvar, vaginal, penile, and oropharyngeal [1,2]. Persistent infection with high-risk HPV types, most notably HPV-16 and HPV-18, accounts for approximately 90% of cervical cancer cases worldwide and contributes substantially to the global burden of HPV-related diseases [3]. The introduction of HPV vaccination has markedly reduced the incidence of HPV-associated precancerous lesions and cancers in populations with high coverage [4,5]. In the United States, the Advisory Committee on Immunization Practices (ACIP) recommends routine HPV vaccination at ages 11–12 years, with catch-up vaccination through age 26 years [6]. Despite these recommendations, national vaccination rates remain below the Healthy People 2030 target of 80% completion among adolescents aged 13–15 years [7].

Data from the National Immunization Survey–Teen (NIS-Teen) have consistently shown persistent differences in HPV vaccination by age and sex, with females and older adolescents more likely to complete the vaccine series, defined as receipt of all ACIP-recommended doses for age. Analyses of NIS-Teen data from 2018–2022 reported completion rates ranging from approximately 50% to 60% among adolescents, with consistently higher completion among females and older age groups [8,9]. Monitoring such trends is essential for assessing progress toward national immunization goals and identifying opportunities for programmatic improvement.

To inform these efforts, the present analysis examines national trends in HPV vaccination uptake among U.S. adolescents aged 13–17 years from 2021 through 2023 using weighted data from the NIS-Teen survey. Specifically, the study describes the percentage of adolescents who initiated and completed the HPV vaccination series across the most recent available adolescent vaccination data, survey years 2021–2023, stratified by age group and sex at birth. Understanding these patterns provides a current benchmark for evaluating national progress toward full HPV vaccine coverage and is especially important given recent reports indicating that HPV vaccination rates have largely stalled nationally, even as uptake of other adolescent vaccines continues to improve [10]. Thus, even with more recent policy reports, the latest available NIS-Teen data offer essential, up-to-date insight into real-world HPV vaccination patterns. Prior research and national policy reports also highlight the need for ongoing, high-quality monitoring of adolescent immunization trends to identify emerging gaps, support timely intervention, and track progress toward national goals [11–13]. Continued surveillance of initiation and completion patterns is therefore essential for guiding future public health strategies aimed at increasing series completion and reducing vaccine-preventable HPV-related cancers.

## Methods

### Study design and data source

This study is a descriptive, repeated cross‑sectional secondary analysis of data from the 2021–2023 National Immunization Survey–Teen (NIS‑Teen) public‑use files. The NIS-Teen is an annual, nationally representative cross-sectional survey conducted by the U.S. Centers for Disease Control and Prevention (CDC) to monitor vaccination coverage among adolescents aged 13–17 years across all 50 states and the District of Columbia. The survey uses a stratified, random-digit–dial sampling design with clustering at the household level. In each survey year, parents or guardians complete a telephone interview, after which permission is requested to contact all identified vaccination providers. Providers then submit verified vaccination histories. Household response rates were 21.0% in 2021, 23.0% in 2022, and 20.7% in 2023, with adequate provider data obtained for approximately 42%–45% of interviewed adolescents each year. These provider-verified data produce weighted national estimates that account for the survey’s complex sampling design, nonresponse adjustments, and household selection probabilities, thereby enabling generalization to the U.S. adolescent population [13].

### Study population

The analytic sample included 126,226 adolescents aged 13–17 years with provider‑verified HPV vaccination data from the 2021–2023 National Immunization Survey–Teen (NIS‑Teen) public‑use files (Table 1). Adolescents with missing or unknown HPV dose data were excluded by the investigators for analytic purposes, yielding an unweighted sample size of 126,226. CDC-supplied sampling weights were applied to generate nationally representative estimates.

**Table 1:** Characteristics of Participants (Weighted and Unweighted) for 2021–2023 (n = 126,226).

Characteristic	n	Unweighted %	Weighted %
Age groups			
13–14	49,596	39.3%	39.8%
15–17	76,630	60.7%	60.2%
Sex at Birth			
Male	65,899	52.2%	51.2%
Female	60,327	47.8%	48.8%

*Note.* Weighted estimates account for the survey’s complex sampling design

The NIS-Teen public-use files include provider-reported vaccination histories but do not contain CDC’s internal harmonized dose-validation variables used for official surveillance reporting (e.g., “up-to-date” indicators that incorporate dose-interval adjudication and multi-provider reconciliation). As a result, HPV vaccination completion in this analysis was operationalized by the investigators using dose-count variables available in the public-use files in conjunction with ACIP age-based dosing recommendations [6]. Estimates derived from this approach may differ from CDC’s internally reported “up-to-date” indicators published in annual surveillance reports.

### Measures

HPV vaccination status was derived from provider‑reported dose variables available in the NIS‑Teen public‑use files. The primary outcome was HPV vaccination status, categorized into three mutually exclusive groups: (1) no initiation (receipt of zero HPV doses), (2) initiated but not completed, and (3) series completion.

Dose counts were identified using the public‑use variable HPVDOSE. Age at series initiation was determined using the reported age at first HPV dose (HPVAGE1). Per Advisory Committee on Immunization Practices (ACIP) guidelines, adolescents initiating the HPV vaccine series before their 15th birthday were classified as complete after receipt of two doses administered at least five months apart, whereas those initiating at age 15 years or older were classified as complete after receipt of three doses administered according to the recommended schedule (0, 1–2, and 6 months) [6].

Adolescents with dose counts that did not meet minimum ACIP interval criteria or with incomplete timing information were classified as “initiated but not completed.” Immunocompromised status could not be identified in the public‑use files and thus could not be incorporated into dose adjudication.

### Statistical analysis

All analyses were descriptive in nature. No formal statistical hypothesis testing, regression modeling, or trend testing was conducted. Instead, weighted frequencies, percentages, and corresponding 95% confidence intervals (CIs) were calculated to describe HPV vaccination initiation and completion patterns across survey years, age groups, and sex at birth.

Survey weights supplied by the CDC (PROVWT) were applied to account for the NIS‑Teen’s complex sampling design, including stratification, clustering, and nonresponse adjustments. Estimates were generated using the Complex Samples module in IBM SPSS Statistics (Version 29).

Stability and differences across years and subgroups were assessed by comparing point estimates and the degree of overlap between 95% confidence intervals, consistent with standard descriptive surveillance reporting practices.

## Results

### Sample characteristics

The analytic sample included 126,226 adolescents aged 13–17 years with provider-verified HPV vaccination data from the 2021–2023 NIS-Teen datasets. Weighted estimates indicated that 39.8% of adolescents were aged 13–14 years and 60.2% were aged 15–17 years; 51.2% were male and 48.8% female (Table 1).

### HPV vaccination coverage by year

Across the 2021–2023 survey years, HPV vaccination status among U.S. adolescents showed limited year-to-year variation (Table 2). In 2021, 62.4% of adolescents had completed the HPV vaccine series according to age-specific ACIP recommendations [6], 14.7% had initiated vaccination but not completed the series, and 22.9% had not initiated HPV vaccination.

**Table 2:** Weighted HPV Vaccination Status by Age Group, Sex at Birth, and Survey Year.

Age Group	Sex at Birth	Survey Year	Did Not Initiate, % (95% CI)	Initiated Not Completed, % (95% CI)	Completed, % (95% CI)
13–14	Male	2021	28.8 (26.1–31.6)	17.4 (15.4–19.6)	53.9 (50.8–56.9)
		2022	28.9 (26.4–31.7)	15.9 (14.0–18.1)	55.1 (52.3–57.9)
		2023	29.1 (26.2–32.1)	17.4 (15.0–20.0)	53.5 (50.4–56.7)
	Female	2021	25.3 (22.6–28.2)	18.2 (16.0–20.8)	56.4 (53.3–59.6)
		2022	22.7 (20.3–25.4)	18.6 (16.3–21.0)	58.7 (55.7–61.6)
		2023	23.9 (21.4–26.6)	18.5 (16.0–21.3)	57.6 (54.4–60.8)
15–17	Male	2021	23.2 (21.0–25.5)	12.0 (10.5–13.7)	64.8 (62.3–67.3)
		2022	21.0 (19.0–23.0)	12.4 (11.0–13.9)	66.7 (64.4–68.9)
		2023	21.6 (19.5–23.9)	12.8 (11.1–14.8)	65.6 (63.0–68.1)
	Female	2021	16.3 (14.2–18.7)	13.1 (11.3–15.0)	70.6 (68.0–73.2)
		2022	16.6 (14.8–18.7)	12.5 (10.8–14.3)	70.9 (68.5–73.2)
		2023	16.7 (14.9–18.6)	13.9 (12.0–16.0)	69.5 (66.9–71.9)

*Note.* Estimates are weighted using NIS‑Teen provider weights. This table presents descriptive estimates (percentages and 95% confidence intervals); no formal hypothesis testing was conducted.

In 2022, series completion was 63.9%, the proportion who had initiated but not completed vaccination was 14.4%, and non-initiation was 21.7%. In 2023, series completion was 62.5%, initiation without completion was 15.2%, and non-initiation was 22.2%. Across all three survey years, approximately one-fifth of adolescents had not initiated HPV vaccination, while roughly one in seven had initiated vaccination without completing the recommended series (Table 1).

### Differences by age group and sex

Consistent differences in HPV vaccination status were observed by age group and sex at birth across all survey years (Table 2). Adolescents aged 15–17 years had higher HPV vaccine series completion than adolescents aged 13–14 years in each year examined. Females also demonstrated higher completion rates than males within both age groups.

Among adolescents aged 13–14 years, series completion ranged from 53.5% to 55.1% among males and from 56.4% to 58.7% among females across 2021–2023. Initiation without completion was more common in this age group, and non-initiation was highest among 13–14-year-old males.

Among adolescents aged 15–17 years, series completion ranged from 64.8% to 66.7% among males and from 69.5% to 70.9% among females across survey years. Although completion was higher among older adolescents, partial vaccination and non-initiation were observed across all subgroups.

### Overall trends

**Fig 1:**
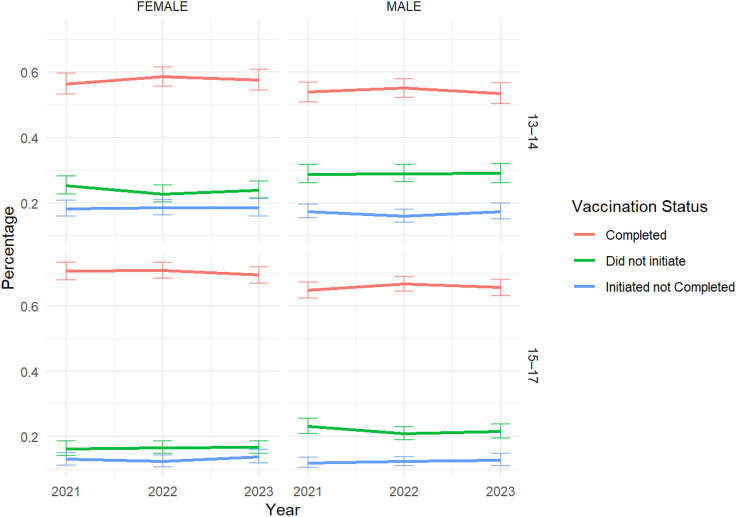
HPV vaccination status trends among adolescents aged 13–17, 2021–2023. Weighted percentages are shown by age group (13–14, 15–17), sex (female, male), and year (2021–2023). Lines represent vaccination status categories: completed, did not initiate, and initiated but not completed. Error bars indicate confidence intervals.

Overall, HPV vaccination patterns among U.S. adolescents demonstrated minimal variation from 2021 through 2023, with series completion estimates ranging between 62.4% and 63.9%. Although these findings suggest stability during this period, the analysis is descriptive and does not formally test temporal trends. Furthermore, the study period coincides with the COVID‑19 recovery phase, during which preventive healthcare utilization was disrupted. As such, observed stability may plausibly reflect maintenance of existing vaccination coverage during a period of healthcare system strain rather than a definitive long‑term plateau in HPV vaccine uptake (Fig 1).

## Discussion

This national analysis of NIS-Teen data indicates that HPV vaccine series completion among U.S. adolescents remained stable from 2021 through 2023, with overall completion estimates ranging from approximately 62% to 64%. Despite this stability, substantial gaps in HPV vaccination persist. Approximately one in five adolescents remained unvaccinated each year, and an additional one in seven initiated the vaccine series without completing it. Completion was consistently higher among older adolescents (15–17 years) than younger adolescents (13–14 years) and higher among females than males. Although completion did not decline during this period, the absence of sustained improvement suggests ongoing challenges in translating vaccine initiation into full series adherence. Importantly, this three-year analytic window coincides with the COVID-19 recovery period, during which healthcare access and preventive service utilization were disrupted. As such, observed stability may reflect preservation of vaccination coverage during a period of system strain rather than long-term stagnation in HPV vaccine uptake.

Age-related trends also provide insight into opportunities for improvement. Older adolescents (15–17 years) exhibited higher completion rates, likely reflecting increased time since vaccine eligibility and additional opportunities for healthcare encounters. However, delaying initiation until mid-adolescence leaves less time to complete the series before the onset of sexual activity, reducing the preventive potential of vaccination [6]. In addition, adolescents who initiate HPV vaccination before age 15 require only a 2-dose series, whereas those initiating at age 15 years or older require a 3-dose series, increasing the complexity of completion for older initiators. Early initiation, ideally at 11–12 years, therefore remains a strong predictor of series completion, emphasizing the importance of provider engagement during routine pediatric and middle-school visits [14,15]. These results indicate that while most adolescents initiate the vaccination series, a consistent subset fails to complete it, potentially due to scheduling barriers, waning parental motivation, or insufficient provider follow-up reminders.

Although the present analysis focused solely on describing age- and sex-specific trends, prior literature demonstrates that HPV vaccination uptake is also shaped by broader sociodemographic and contextual factors, including race and ethnicity, household income, parental education, and healthcare access [16,17]. The current trend analysis did not examine these factors, and therefore no conclusions can be drawn about their influence within this dataset. However, previous studies consistently show that persistent non-initiation and lower completion rates often occur in populations facing structural or access-related barriers, such as adolescents living in rural areas or lower-income households [18,19]. While these factors were not evaluated in the present study, they provide important context for interpreting national patterns and underscore the need to explore multilevel contributors to HPV vaccination uptake in future analyses. Building on these descriptive findings, future analyses will assess how sociodemographic and healthcare access factors shape HPV vaccination initiation and completion.

From a public health perspective, the continued stability in HPV vaccination completion rates underscores the need for renewed, system-level interventions. Proven strategies, such as standing orders in primary care pediatric clinics, school‑based vaccination programs, text‑message reminder systems, immunization information system (IIS)–based recalls, and electronic health record prompts, remain essential for improving series completion and should be expanded and evaluated for scalability [20,21]. Strengthening provider communication and addressing parental vaccine hesitancy through targeted training may also enhance adherence to recommended vaccination schedules [22]. Recent national immunization data reinforce this urgency, with the CDC’s 2025 adolescent vaccination coverage report showing improvement in other routine adolescent vaccines while HPV vaccination coverage remains unchanged [10]. Updated recommendations from the American Academy of Pediatrics similarly emphasize consistent preventive-care visits and adolescent vaccination platforms, which may increase opportunities to deliver multi-dose vaccines such as [11]. In addition, emerging federal discussions related to vaccine oversight, exemptions, and preventive-care infrastructure may influence future adolescent vaccination patterns. Although these broader policy developments were not evaluated in this analysis, they highlight the importance of continued surveillance as policy environments evolve. These considerations align with broader federal priorities outlined in the *Vaccines National Strategic Plan 2021–2025*, which emphasizes strengthening immunization infrastructure through improved data interoperability, enhanced equity strategies, and more robust monitoring systems [13]. Collectively, these public health and policy priorities underscore the need to reinforce national monitoring and delivery systems to support not only initiation but also completion of the HPV vaccine series.

This study has several limitations. First, HPV vaccination estimates derived from the public-use NIS-Teen dataset may differ from those reported in CDC’s annual Morbidity and Mortality Weekly Report (MMWR) publications [23]. The public-use files do not contain CDC’s internal harmonized dose-validation variables, imputation procedures, or total survey error (TSE) adjustments used in official surveillance reporting. As a result, the strict provider-verified completion measure used here is expected to yield more conservative (lower) estimates than CDC’s “up-to-date” indicators. These discrepancies reflect methodological differences rather than issues with data quality. Second, only three years of data were analyzed (2021–2023), limiting our ability to infer long-term trends beyond short-term stability during the COVID-19 recovery period. Third, this study was descriptive and did not examine sociodemographic or contextual predictors of uptake. Future work comparing Public Use Files (PUF) based completion estimates with CDC internal Up-to_date (UTD) measures may help clarify the impact of dose-validation and TSE procedures on HPV coverage estimates.

## Conclusion

HPV vaccination among U.S. adolescents aged 13–17 years has remained largely stable from 2021 through 2023, with females and older adolescents demonstrating modestly higher completion rates. Despite national initiatives and clinical guidelines promoting routine vaccination, coverage continues to fall short of the Healthy People 2030 target. These findings reinforce the need for early vaccination, consistent provider recommendations, and policy-driven interventions to support completion of the HPV vaccine series. Integrating sociodemographic analyses in future research will be critical to identifying persistent disparities and advancing equitable progress in HPV-related cancer prevention.
