# Supplementary material for: Trends in HPV vaccination initiation and completion among U.S. adolescents aged 13–17 years, 2021–2023: Findings from the National Immunization Survey–Teen
Source: PLoS One. 2026 Jun 8;21(6):e0349299. doi: 10.1371/journal.pone.0349299 (PMC13245753; doi:10.1371/journal.pone.0349299)
Supplement: Attachment — Submitted filename: PLOS One Review Letter.docx [file pone.0349299.s001.docx]

**Taylor Mullin Ph.D., RN, CNOR**

**The Ohio State University College of Nursing**

317 Newton Hall
295 W. 10th Avenue, Columbus, OH 43210
614-404-3585
[mullin.94@osu.edu](mailto:mullin.94@osu.edu)

April 23, 2026

**Editorial Office
PLOS ONE**

**Manuscript ID: PONE‑D‑26‑11243R1**

Dear Editor and Reviewers,

Thank you for your thoughtful review and invaluable comments. We believe the revised version, based on your input, improved the quality of this paper. Our point-by-point responses to the specific comments from the reviewers are listed below. We also highlighted the changes we made in the revised version of the manuscript specific to the responses to reviewers’ comments.

**Editor:**

We sincerely thank the Editor for the thoughtful evaluation of our manuscript and for the clear guidance provided. We have addressed all requested revisions to strengthen methodological clarity, transparency, reproducibility, and data presentation in accordance with PLOS ONE standards.

Specifically, we have (1) explicitly clarified the analytical approach as descriptive and stated that no formal statistical hypothesis testing was conducted (Methods, Statistical Analysis; ~lines 210–225); (2) expanded the Methods section to provide detailed, step‑by‑step descriptions of variable definitions, ACIP dosing algorithms, dose adjudication, handling of invalid schedules, and the specific survey weight applied (Methods, Measures and Statistical Analysis; ~lines 170–245); (3) moved the former Supplemental Table 1 into the main manuscript as an embedded table presenting weighted estimates with 95% confidence intervals and an explanatory footnote (Results, Tables 1 and 2; ~lines 255–360); (4) refined the Discussion to remove duplicated content, temper speculative language related to COVID‑19 recovery, expand interpretation of the “initiated but not completed” group, and strengthen policy implications with specific, actionable recommendations (Discussion; ~lines 430–560); and (5) corrected all editorial and formatting issues, including reference formatting, declarations, and removal of non‑standard manuscript elements.

We believe these revisions substantially improve the manuscript’s clarity, transparency, and alignment with PLOS ONE’s criteria for methodological soundness and reproducibility.

**Reviewer #1:**

We sincerely thank Reviewer 1 for the detailed, constructive, and rigorous review. We greatly appreciate the emphasis on methodological transparency and reproducibility, which has substantially strengthened the manuscript. Each comment is addressed below.

**Comment 1:** The statistical approach is not clearly specified; “all analyses” is vague.
**Response:** We revised the Statistical Analysis subsection to explicitly describe all analyses conducted. The manuscript now clearly states that the study is descriptive in nature and that weighted frequencies, percentages, and 95% confidence intervals were calculated. We explicitly note that no formal hypothesis testing, regression modeling, or trend testing was performed (Methods, Statistical Analysis; ~lines 215–225).

**Comment 2:** Data source and access are unclear.
**Response:** We clarified that data were obtained from the CDC‑maintained National Immunization Survey–Teen (NIS‑Teen) public‑use files and described the access source and years analyzed in the Study Design and Data Source section (Methods; ~lines 120–155).

**Comment 3–4:** Overuse and ambiguity of the term “complex”; analyses appear descriptive.
**Response:** We revised language throughout the manuscript to consistently describe the study as a descriptive, repeated cross‑sectional analysis. References to “complex” are now limited to describing the survey design rather than analytical complexity (Methods and Discussion; multiple locations).

**Comment 5:** Significance testing and thresholds not described.
**Response:** We now explicitly state that no statistical significance testing was performed and that interpretations are based on descriptive comparison of point estimates and confidence intervals (Methods, Statistical Analysis; ~lines 215–225).

**Comment 6:** Figure 1 methods and software not specified.
**Response:** We clarified that Figure 1 was generated using IBM SPSS Statistics (Version 29) based on weighted estimates. Figure resolution was also improved to ensure legibility (Methods; ~lines 225–230).

**Comment 7:** Reproducibility requires a step‑by‑step description of methods.
**Response:** The Measures and Statistical Analysis sections were substantially expanded to include exact variable names (e.g., HPVDOSE, HPVAGE1), ACIP 2‑dose versus 3‑dose rules, minimum interval considerations, handling of invalid or incomplete schedules, inability to identify immunocompromised status, and the exact survey weight used (PROVWT), enabling full replication (Methods; ~lines 170–245).

**Comment 8:** Overstatement of “trends” and “stability.”
**Response:** We reframed the manuscript to avoid implying formal trend testing and consistently describe findings as descriptive. All references to “stability” are now interpreted within the context of overlapping confidence intervals, and speculative language has been tempered (Results and Discussion; ~lines 300–360 and 430–470).

**Comment 9:** Series‑completion definition lacks sufficient detail.
**Response:** We expanded the Measures section to fully describe dose‑count variables, age at initiation determination, ACIP schedule application, interpretation of invalid schedules, and limitations related to unavailable CDC internal harmonized UTD indicators (Methods; ~lines 170–210).

**Comment 10:** Supplemental table should be moved to the main text with CIs.
**Response:** The former Supplemental Table 1 has been moved into the main manuscript as Table 2, presenting weighted percentages and 95% confidence intervals for vaccination status by age group, sex at birth, and survey year. An explanatory footnote clarifies that the analysis is descriptive (Results; Table 2; ~lines 300–360).

**Comment 11:** Discussion contains duplication and over‑interpretation of COVID recovery.
**Response:** The duplicated Discussion paragraph was removed entirely. Remaining language now frames the COVID‑19 recovery explanation as a plausible hypothesis rather than a causal conclusion (Discussion; ~lines 440–470).

**Comment 12:** References require cleanup and Vancouver formatting.
**Response:** All references were reformatted to Vancouver style; duplicate and tertiary sources were removed where possible; incomplete citations were corrected; and DOIs were added when available (References; ~lines 610–end).

**Minor Comments**

All minor comments were addressed, including:

- Describing the study throughout as a repeated cross‑sectional secondary analysis
- Adding methods and setting details to the abstract
- Including exact percentages alongside qualitative descriptors (e.g., “one in five (22.2%)”)
- Removing author biographies and correcting declarations to plural language
- Spelling out abbreviations (MMWR, UTD, PUF) at first mention
- Resolving all line‑specific editorial and formatting issues noted in the annotated manuscript

**Response to Reviewer 2**

We thank Reviewer 2 for the positive evaluation of the manuscript and for recognizing the public health relevance and practical implications of this work. No changes were requested.

We are grateful to the Academic Editor and reviewers for their insightful feedback. We believe these revisions have substantially strengthened the manuscript and improved its transparency, reproducibility, and alignment with PLOS ONE standards. We appreciate the opportunity to revise and resubmit and look forward to further consideration.

Sincerely,

Taylor Mullin

**Taylor Mullin Ph.D., RN, CNOR**

[Mullin.94@osu.edu](mailto:Mullin.94@osu.edu)

**614-404-3585**
