# Supplementary material for: Trends in HPV vaccination initiation and completion among U.S. adolescents aged 13–17 years, 2021–2023: Findings from the National Immunization Survey–Teen
Source: PLoS One. 2026 Jun 8;21(6):e0349299. doi: 10.1371/journal.pone.0349299 (PMC13245753; doi:10.1371/journal.pone.0349299)
Supplement: Peer review history

# Peer review: Decision Letter 0

**DOI:** 10.1371/journal.pone.0349299.r001

**Authors:** Morufu Olalekan Raimi

19 Mar 2026

PONE-D-26-11243

Trends in HPV Vaccination Initiation and Completion Among U.S. Adolescents Aged 13–17 Years, 2021–2023: Findings from the National Immunization Survey–Teen

PLOS One

Dear Dr. Mullin,

Thank you for submitting your manuscript to PLOS ONE. After careful consideration, we feel that it has merit but does not fully meet PLOS ONE’s publication criteria as it currently stands. Therefore, we invite you to submit a revised version of the manuscript that addresses the points raised during the review process.

Please submit your revised manuscript by May 03 2026 11:59PM. If you will need more time than this to complete your revisions, please reply to this message or contact the journal office at plosone@plos.org. When you're ready to submit your revision, log on to https://www.editorialmanager.com/pone/ and select the 'Submissions Needing Revision' folder to locate your manuscript file.

Please include the following items when submitting your revised manuscript:

- A letter that responds to each point raised by the academic editor and reviewer(s). You should upload this letter as a separate file labeled 'Response to Reviewers'.
- A marked-up copy of your manuscript that highlights changes made to the original version. You should upload this as a separate file labeled 'Revised Manuscript with Track Changes'.
- An unmarked version of your revised paper without tracked changes. You should upload this as a separate file labeled 'Manuscript'.

-->If you would like to make changes to your financial disclosure, please include your updated statement in your cover letter. Guidelines for resubmitting your figure files are available below the reviewer comments at the end of this letter.

If applicable, we recommend that you deposit your laboratory protocols in protocols.io to enhance the reproducibility of your results. Protocols.io assigns your protocol its own identifier (DOI) so that it can be cited independently in the future. For instructions see: https://journals.plos.org/plosone/s/submission-guidelines#loc-laboratory-protocols. Additionally, PLOS ONE offers an option for publishing peer-reviewed Lab Protocol articles, which describe protocols hosted on protocols.io. Read more information on sharing protocols at https://plos.org/protocols?utm_medium=editorial-email&utm_source=authorletters&utm_campaign=protocols.

We look forward to receiving your revised manuscript.

Kind regards,

Morufu Olalekan Raimi, Ph.D

Academic Editor

PLOS One

Journal Requirements:

When submitting your revision, we need you to address these additional requirements.

1. Please ensure that your manuscript meets PLOS ONE's style requirements, including those for file naming. The PLOS ONE style templates can be found at

https://journals.plos.org/plosone/s/file?id=wjVg/PLOSOne_formatting_sample_main_body.pdf and

https://journals.plos.org/plosone/s/file?id=ba62/PLOSOne_formatting_sample_title_authors_affiliations.pdf

2. We note that the grant information you provided in the ‘Funding Information’ and ‘Financial Disclosure’ sections do not match.

When you resubmit, please ensure that you provide the correct grant numbers for the awards you received for your study in the ‘Funding Information’ section.

3. Thank you for stating the following financial disclosure:

“Midwest Nursing Research Society Advocate Health Dissertation Grant”

Please state what role the funders took in the study. If the funders had no role, please state: "The funders had no role in study design, data collection and analysis, decision to publish, or preparation of the manuscript."

If this statement is not correct you must amend it as needed.

Please include this amended Role of Funder statement in your cover letter; we will change the online submission form on your behalf.

4. Thank you for stating the following in your Competing Interests section:

“NO authors have competing interests”

Please complete your Competing Interests on the online submission form to state any Competing Interests. If you have no competing interests, please state "The authors have declared that no competing interests exist.", as detailed online in our guide for authors at http://journals.plos.org/plosone/s/submit-now

This information should be included in your cover letter; we will change the online submission form on your behalf.

5. Please include your tables as part of your main manuscript and remove the individual files. Please note that supplementary tables (should remain/ be uploaded) as separate "supporting information" files.

6. Please include captions for your Supporting Information files at the end of your manuscript, and update any in-text citations to match accordingly. Please see our Supporting Information guidelines for more information: http://journals.plos.org/plosone/s/supporting-information.

7. If the reviewer comments include a recommendation to cite specific previously published works, please review and evaluate these publications to determine whether they are relevant and should be cited. There is no requirement to cite these works unless the editor has indicated otherwise.

8. Please review your reference list to ensure that it is complete and correct. If you have cited papers that have been retracted, please include the rationale for doing so in the manuscript text, or remove these references and replace them with relevant current references. Any changes to the reference list should be mentioned in the rebuttal letter that accompanies your revised manuscript. If you need to cite a retracted article, indicate the article’s retracted status in the References list and also include a citation and full reference for the retraction notice.

Additional Editor Comments:

Date: March 19, 2026

Manuscript Number: PONE-D-26-11243R1

Title: Trends in HPV Vaccination Initiation and Completion Among U.S. Adolescents Aged 13–17 Years, 2021–2023: Findings from the National Immunization Survey–Teen

Corresponding Author: Taylor Mullin, MSN, RN, CNOR

Decision: Minor Revision

Dear Ms. Mullin,

Thank you for submitting your revised manuscript, “Trends in HPV Vaccination Initiation and Completion Among U.S. Adolescents Aged 13–17 Years, 2021-2023: Findings from the National Immunization Survey-Teen,” for consideration for publication in PLOS ONE. I have now received the comments from the original reviewers and have conducted a thorough re-evaluation of your work in the context of their feedback.

First, I want to commend you on the quality and public health significance of your research. This study provides timely and essential surveillance data on HPV vaccination trends during a critical post-pandemic period. The use of the nationally representative NIS-Teen dataset, the clear delineation of initiation and completion, and the stratification by age and sex provide a valuable benchmark for policymakers and clinicians. Your interpretation of the findings, particularly the cautious framing of "stability" within the context of healthcare disruption, is both appropriate and insightful.

As you can see from the attached comments, the reviews are largely positive. However, the critiques raised by Reviewers 1 and 3, which focus on methodological rigor and reproducibility, are substantial and must be addressed to bring the manuscript in line with PLOS ONE's standards for methodological soundness. Review 2 was unconditionally positive, but we must prioritize the more detailed, constructive critiques to ensure the published work is as robust and transparent as possible.

Based on the consensus of the reviews and my own assessment, I am pleased to offer you the opportunity to revise your manuscript. The required revisions are considered "minor" in scope, but they are critical for strengthening the paper's clarity, transparency, and overall impact. The primary areas requiring your attention are:

Essential Revisions:

1. Clarify the Analytical Approach (Addressing Reviewer 1 & 3): The most significant revision involves clearly defining your analysis as a descriptive study. You must explicitly state that no formal statistical hypothesis testing (e.g., for trends over time or differences between groups) was performed. Please revise the Statistical Analysis subsection of your Methods to state this plainly. You can note that the analysis relied on comparing weighted percentages and their confidence intervals to assess the stability and differences in estimates, which is an appropriate descriptive approach. This will directly address the concerns raised by both reviewers about overstating "trends" without supporting statistical tests.

2. Enhance Methodological Reproducibility (Addressing Reviewer 1 & 3): To allow other researchers to replicate your work, you must provide a step-by-step account of your methodology. Please expand the Measures and Statistical Analysis sections to include:

o The exact variable names from the NIS-Teen public-use files that were used (e.g., HPVDOSE or similar).

o The specific algorithm used to determine age at initiation and how you operationalized the ACIP 2-dose vs. 3-dose rules (e.g., how you handled dose timing and intervals).

o A statement on how adolescents with invalid dose schedules (if any) were handled.

o The exact name of the weight variable applied (e.g., PROVWT).

3. Complete Data Presentation (Addressing Reviewer 1 & 3): A descriptive study must present its data fully to allow readers to draw their own conclusions.

o Please move the data from Supplemental Table 1 into the main manuscript as a new Table 1. This table should present the weighted percentages for each vaccination status category (non-initiation, initiation without completion, completion) for the total sample and each subgroup (age, sex), with their corresponding 95% Confidence Intervals for each survey year. This will allow readers to immediately assess the precision of your estimates and the degree of overlap between years and groups.

o Add a concise footnote to the table explaining that estimates are weighted and that the analysis is descriptive, focusing on the comparison of point estimates and confidence intervals.

o Please ensure Figure 1 is of high resolution and clearly legible.

4. Refine and Focus the Discussion (Addressing Reviewer 1 & 3):

o Eliminate Duplication: Remove the repeated paragraph on the “stability during COVID-19” point. Consolidate this discussion into one concise, well-argued section.

o Deepen the Interpretation: Expand the discussion on the “initiated but not completed” group. What are the public health implications? What known barriers (e.g., logistical, motivational) might this group face compared to the “never initiated”?

o Strengthen Policy Implications: Reviewer 3 correctly notes the recommendations are generic. Please make them more specific. For example, instead of “standing orders,” specify “standing orders in primary care pediatric clinics." Instead of "reminder-recall systems,” mention effective modalities like “text message reminders or immunization information system (IIS)-based recalls.”

o Temper Speculative Language: Frame the explanation regarding the COVID-19 recovery period as a plausible hypothesis for the observed stability, not as a definitive conclusion derived from your data. This aligns with Reviewer 1's astute observation.

5. Correct Editorial and Formatting Issues:

o References: Please conduct a thorough review of your reference list. Correct the formatting to the Vancouver style as per PLOS ONE guidelines. Eliminate duplicate entries, complete any incomplete references (adding DOIs where available), and ensure all in-text citations have a corresponding entry in the list.

o Author Bios: Please remove the “Author Bios” section from the end of the manuscript, as this is not standard for a research article in PLOS ONE. This information is more suitable for a cover letter or a separate biographical statement if requested.

o Declaration of Interest: Correct the singular “The author declares...” to the plural “The authors declare no competing interests.”

o In-Text Percentages: When describing proportions like “one in five,” please add the exact percentage in parentheses (e.g., “one in five (22.2%)...”).

We look forward to receiving your revised manuscript. When you resubmit, please include a detailed, point-by-point response to the reviews, outlining the changes you have made and citing the relevant line numbers in the revised manuscript. This will expedite the re-review process.

I am confident that with these revisions, your manuscript will make a strong and valuable contribution to the literature on adolescent immunization.

Sincerely,

Dr. Morufu Olalekan Raimi, PhD

Editor, PLOS ONE

[Note: HTML markup is below. Please do not edit.]

Reviewers' comments:

Reviewer's Responses to Questions

**Comments to the Author**

1. Is the manuscript technically sound, and do the data support the conclusions?

The manuscript must describe a technically sound piece of scientific research with data that supports the conclusions. Experiments must have been conducted rigorously, with appropriate controls, replication, and sample sizes. The conclusions must be drawn appropriately based on the data presented.

Reviewer #1: No

Reviewer #2: Yes

**********

2. Has the statistical analysis been performed appropriately and rigorously?

Reviewer #1: No

Reviewer #2: Yes

**********

3. Have the authors made all data underlying the findings in their manuscript fully available?

The PLOS Data policy requires authors to make all data underlying the findings described in their manuscript fully available without restriction, with rare exception (please refer to the Data Availability Statement in the manuscript PDF file). The data should be provided as part of the manuscript or its supporting information, or deposited to a public repository. For example, in addition to summary statistics, the data points behind means, medians and variance measures should be available. If there are restrictions on publicly sharing data—e.g. participant privacy or use of data from a third party—those must be specified.

Reviewer #1: Yes

Reviewer #2: Yes

**********

4. Is the manuscript presented in an intelligible fashion and written in standard English?

PLOS ONE does not copyedit accepted manuscripts, so the language in submitted articles must be clear, correct, and unambiguous. Any typographical or grammatical errors should be corrected at revision, so please note any specific errors here.

Reviewer #1: Yes

Reviewer #2: Yes

**********

5. Review Comments to the Author

Please use the space provided to explain your answers to the questions above. You may also include additional comments for the author, including concerns about dual publication, research ethics, or publication ethics. (Please upload your review as an attachment if it exceeds 20,000 characters)

Reviewer #1: Dear Authors and Editor,

Thank you for the opportunity to peer-review the present manuscript. I myself learned quite a lot from the manuscript and found the presentation quite interesting. The manuscript presents a useful descriptive analysis of recent NIS-teen public-use data on HPV initiation and completion (an important analysis considering the need to complete vaccination series). I believe the scope is appropriate for PLOS ONE, but I don’t believe the paper is ready yet to be published with PLOS ONE considering the journal’s aim of methodological soundness, which I believe this paper had quite key yet significant limitations with. The paper’s descriptive message is reasonable, but the manuscript still needs tighter analytic framing, fuller methodological reproducibility, and substantial cleanup before it meets PLOS ONE’s standard that analyses be described in sufficient detail and conclusions be supported by the data.

My comments are offered in the spirit of constructive scholarly review and should be read as an effort to strengthen the manuscript, not as a harsh judgment of the authors’ work. I appreciate the importance of the study and hope these suggestions are helpful. Regardless of my recommendation, I have tried to be as thorough and constructive in enabling the authors to improve the present manuscript whether it is ultimately published with PLOS ONE or elsewhere.

Critical Comments

The methodology part is probably the weakest portion of the paper. The statistical approach is not detailed thoroughly (I cannot replicate what the authors have done) and it is sort of misleading.

1. You mentioned “All analyses”, but what are the analyses? Can you please specify what analytical approaches were taken?

2. You mentioned that you got the data from the NIS-Teen’s sample, but how can I access this data? Is it from a website? A private database? When was this data accessed by the authors?

3. You mention the word “complex” a lot. It appears that the survey method is “complex” but I would use this word carefully. I am mentioning this as I don’t see any definition or standard of what complex is. E.g., it is complex but according to who?

4. It also appears that the manuscript is describing its methods as complex, which I would be careful of. The analyses present appear to be descriptive in nature, so I would be cautious with wording here. Please make sure to explicitly label your analyses as descriptive.

5. What was significance set to? How did you measure significance? What tools? Etc.

6. I also see data visualization was done with Figure 1. I would like you to explain briefly how it was done and with what software.

7. Overall, what I would like here is a step-by-step guide of how I can replicate your approach to get exactly all the results you have gotten.

Major Comments

• The manuscript overstates “trend” and “stability” relative to the analysis actually performed.

o The methods report only weighted frequencies and percentages using complex-survey procedures in SPSS. I do not see formal year-to-year hypothesis tests, regression modelling, trend tests, or even CIs in the main text. With the supplement, I can see that subgroup estimates are fairly similar across years and many CIs overlap, which is consistent with limited year-to-year variation, but that is still not the same as formally demonstrating “stable trends”. I would ask the authors either to add survey-adjusted comparisons across years/subgroups, with exact p-values and clearly reported variance, or to reframe the paper consistently as a descriptive snapshot of 2021–2023 patterns rather than a trend analysis.

o I would also be cautious about using the word “complex” to describe the present work, as it appears descriptive. I would also refrain from labelling the sampling approach as “complex” as that may raise eyebrows on what complex actually means. Choosing more objective descriptions of the work always suits best.

• The HPV series-completion definition still needs more detail to be reproducible.

o The authors appropriately acknowledge that the NIS-Teen public-use files do not contain CDC’s internal harmonized dose-validation / UTD variables, and that they derived completion from public-use-file dose information plus ACIP rules. That is an important strength in transparency. However, the paper still does not specify the exact variables used, how age at initiation was determined, how the minimum interval rule was operationalized, how potentially invalid 2-dose schedules were handled, whether immunocompromised adolescents could be identified, or exactly which survey weight was used. The manuscript currently says only that “CDC-supplied sampling weights were applied,” which is too vague for replication.

o This builds on further comments I make later on about reproducibility issues. I do not know how labels were made, how cutoffs were determined, how to access the data, and how to replicate the analyses.

• Claims should be backed by statistical analyses rather than inferences.

o I would like the supplementary table to be moved to the main text and for you to explicitly mention confidence intervals in the narrative text. This is important as I see significant claims being made narratively but no statistically significant markers to back-up the claim.

o Please note that this comment is a major limitation of the manuscript at present as it limits your findings.

• The discussion contains clear duplication and some over-interpretation.

o There is a repeated paragraph on the paper’s central “stable during COVID-recovery” point.

o The COVID-recovery explanation is plausible but speculative as the analysis does not include a pre-2021 comparison window or a design that could attribute the pattern to recovery-related preservation of uptake. That language should be shortened and framed as a hypothesis, not as an explanatory conclusion.

• References are need some work-up and are not formatted correctly.

o PLOS ONE guidelines ask that manuscripts include referencing in Vancouver style, which is not present.

o The chosen references may also be strengthened, as including more peer-reviewed work is more suitable than web pages.

Minor Comments

• Since the supplemental table reports only percentages and CIs, I would encourage adding subgroup numerators/denominators or subgroup unweighted n, especially because PLOS asks for clear variance reporting and recommends giving numerators/denominators when percentages are presented.

• The paper would read more accurately if it were described throughout as a repeated cross-sectional secondary analysis of 2021–2023 NIS-Teen public-use data.

• Please describe the methods and settings in the abstract.

• I understand why the findings are important, but I would like you to tell me why. How are the current findings significant for healthcare practitioners, government officials, public health workers, etc. Basically, who can use the results and how.

• I believe the manuscript would also heavily benefit from expanding its talk about vaccine initiation vs completion, with exact quantitative data and an expanded discussion of how this is relevant.

• There are several significant editorial issues that still need cleanup.

o Author biographies are appended after the conclusion, which should not be part of the manuscript body.

o The cover page also uses singular phrasing (“The author acknowledges…” / “The author declares…”) despite multiple authors. All authors need to take responsibility for the present work.

o The reference list appears inconsistently formatted and includes at least one tertiary source (UpToDate), which is not ideal when primary or official sources are available.

Within manuscript comments

Format:

Line within the main manuscript document: the comments I left on Microsoft Word

Line 34: Can you please specify the setting of the sample when you mentioned 126,226 were sampled?

Line 91: There’s a missing space.

Line 165: This is a bit confusing, as there is no “Table 2” anywhere but I understand what the authors are pointing to. I believe moving supplemental table 1 into the main manuscript would strengthen the paper for easy and transparent access of data.

Line 172: If you want to describe quantitative data in this format, please add a bracket with the exact percent (e.g. “approximately one-fifth (19.8%)” etc.) or whatever the actual statistic was. Again, this adds to my last point that the second table being in supplement is hard to access.

Line 175: You mentioned numerical differences but I do not see any anchor to support your claims. For example, if you mention group x is greater than group y, you need to explicitly mention the p values or/and confidence intervals that support such a statement.

Line 176: I don’t see any separate longitudinal analysis that support the use of the word “consistent”. I would be cautious of this claim.

Lines 176 through 179: I would remove this paragraph or mention the exact confidence intervals that support these statements.

Lines 192 through 196: This is a good statement to make. Thank you.

Line 201: 95% CIs or p values please. I know 62-64 is a narrow range and looks stable but I would like the claim statistically backed up in the last sentence.

Line 202 (for “one in five”): Please mention the % in brackets.

Line 204: Add quantitative data please.

Line 207: Here it would be good to mention how many have not completed their series as mentioned earlier.

Lines 212 through 220: This paragraph is a duplicate. Please remove.

Line 235: Good line.

Lines 238 through 294: Can you please spell out the abbreviations MMWR, UTD, and PUF?

T his manuscript addresses an important public health topic and has the foundation of a potentially useful descriptive contribution. However, in its current form, the concerns outlined above regarding analytic framing, methodological transparency, reproducibility, and overall manuscript preparation are substantial enough that I am unable to support publication in PLOS ONE at this time considering the journal’s emphasis on methodology. I nevertheless believe the work has merit, and with careful revision and strengthening (which I hope I was able to help with in terms of my comprehensive, constructive, and detailed feedback), the manuscript could develop into a more rigorous and publishable study, whether for this journal in a future form or elsewhere.

Reviewer #2: This is an excellent and highly relevant public health study. Using a massive, nationally representative dataset like the NIS-Teen makes your findings very reliable.

I appreciate how clearly you laid out the trends from 2021 to 2023. Highlighting that vaccination rates stabilized rather than dropped during the COVID-19 recovery period is a very important insight. Your conclusions regarding the need for better reminder-based follow-ups are practical and strongly supported by your data.

I have no changes to request. Congratulations on a well-written and valuable paper.

**********

6. PLOS authors have the option to publish the peer review history of their article (what does this mean?). If published, this will include your full peer review and any attached files.

If you choose “no”, your identity will remain anonymous but your review may still be made public.

**Do you want your identity to be public for this peer review?** For information about this choice, including consent withdrawal, please see our Privacy Policy.

Reviewer #1: No

Reviewer #2: **Yes:** Ganesh Praneeth Roy Avu

# Author comment: Author response to Decision Letter 1

**DOI:** 10.1371/journal.pone.0349299.r002

26 Apr 2026

Editorial Office

PLOS ONE

Manuscript ID: PONE D 26 11243R1

Dear Editor and Reviewers,

Thank you for your thoughtful review and invaluable comments. We believe the revised version, based on your input, improved the quality of this paper. Our point-by-point responses to the specific comments from the reviewers are listed below. We also highlighted the changes we made in the revised version of the manuscript specific to the responses to reviewers’ comments.

Editor:

We sincerely thank the Editor for the thoughtful evaluation of our manuscript and for the clear guidance provided. We have addressed all requested revisions to strengthen methodological clarity, transparency, reproducibility, and data presentation in accordance with PLOS ONE standards.

Specifically, we have (1) explicitly clarified the analytical approach as descriptive and stated that no formal statistical hypothesis testing was conducted (Methods, Statistical Analysis; ~lines 210–225); (2) expanded the Methods section to provide detailed, step by step descriptions of variable definitions, ACIP dosing algorithms, dose adjudication, handling of invalid schedules, and the specific survey weight applied (Methods, Measures and Statistical Analysis; ~lines 170–245); (3) moved the former Supplemental Table 1 into the main manuscript as an embedded table presenting weighted estimates with 95% confidence intervals and an explanatory footnote (Results, Tables 1 and 2; ~lines 255–360); (4) refined the Discussion to remove duplicated content, temper speculative language related to COVID 19 recovery, expand interpretation of the “initiated but not completed” group, and strengthen policy implications with specific, actionable recommendations (Discussion; ~lines 430–560); and (5) corrected all editorial and formatting issues, including reference formatting, declarations, and removal of non standard manuscript elements.

We believe these revisions substantially improve the manuscript’s clarity, transparency, and alignment with PLOS ONE’s criteria for methodological soundness and reproducibility.

Reviewer #1:

We sincerely thank Reviewer 1 for the detailed, constructive, and rigorous review. We greatly appreciate the emphasis on methodological transparency and reproducibility, which has substantially strengthened the manuscript. Each comment is addressed below.

Comment 1: The statistical approach is not clearly specified; “all analyses” is vague.

Response: We revised the Statistical Analysis subsection to explicitly describe all analyses conducted. The manuscript now clearly states that the study is descriptive in nature and that weighted frequencies, percentages, and 95% confidence intervals were calculated. We explicitly note that no formal hypothesis testing, regression modeling, or trend testing was performed (Methods, Statistical Analysis; ~lines 215–225).

Comment 2: Data source and access are unclear.

Response: We clarified that data were obtained from the CDC maintained National Immunization Survey–Teen (NIS Teen) public use files and described the access source and years analyzed in the Study Design and Data Source section (Methods; ~lines 120–155).

Comment 3–4: Overuse and ambiguity of the term “complex”; analyses appear descriptive.

Response: We revised language throughout the manuscript to consistently describe the study as a descriptive, repeated cross sectional analysis. References to “complex” are now limited to describing the survey design rather than analytical complexity (Methods and Discussion; multiple locations).

Comment 5: Significance testing and thresholds not described.

Response: We now explicitly state that no statistical significance testing was performed and that interpretations are based on descriptive comparison of point estimates and confidence intervals (Methods, Statistical Analysis; ~lines 215–225).

Comment 6: Figure 1 methods and software not specified.

Response: We clarified that Figure 1 was generated using IBM SPSS Statistics (Version 29) based on weighted estimates. Figure resolution was also improved to ensure legibility (Methods; ~lines 225–230).

Comment 7: Reproducibility requires a step by step description of methods.

Response: The Measures and Statistical Analysis sections were substantially expanded to include exact variable names (e.g., HPVDOSE, HPVAGE1), ACIP 2 dose versus 3 dose rules, minimum interval considerations, handling of invalid or incomplete schedules, inability to identify immunocompromised status, and the exact survey weight used (PROVWT), enabling full replication (Methods; ~lines 170–245).

Comment 8: Overstatement of “trends” and “stability.”

Response: We reframed the manuscript to avoid implying formal trend testing and consistently describe findings as descriptive. All references to “stability” are now interpreted within the context of overlapping confidence intervals, and speculative language has been tempered (Results and Discussion; ~lines 300–360 and 430–470).

Comment 9: Series completion definition lacks sufficient detail.

Response: We expanded the Measures section to fully describe dose count variables, age at initiation determination, ACIP schedule application, interpretation of invalid schedules, and limitations related to unavailable CDC internal harmonized UTD indicators (Methods; ~lines 170–210).

Comment 10: Supplemental table should be moved to the main text with CIs.

Response: The former Supplemental Table 1 has been moved into the main manuscript as Table 2, presenting weighted percentages and 95% confidence intervals for vaccination status by age group, sex at birth, and survey year. An explanatory footnote clarifies that the analysis is descriptive (Results; Table 2; ~lines 300–360).

Comment 11: Discussion contains duplication and over interpretation of COVID recovery.

Response: The duplicated Discussion paragraph was removed entirely. Remaining language now frames the COVID 19 recovery explanation as a plausible hypothesis rather than a causal conclusion (Discussion; ~lines 440–470).

Comment 12: References require cleanup and Vancouver formatting.

Response: All references were reformatted to Vancouver style; duplicate and tertiary sources were removed where possible; incomplete citations were corrected; and DOIs were added when available (References; ~lines 610–end).

Minor Comments

All minor comments were addressed, including:

• Describing the study throughout as a repeated cross sectional secondary analysis

• Adding methods and setting details to the abstract

• Including exact percentages alongside qualitative descriptors (e.g., “one in five (22.2%)”)

• Removing author biographies and correcting declarations to plural language

• Spelling out abbreviations (MMWR, UTD, PUF) at first mention

• Resolving all line specific editorial and formatting issues noted in the annotated manuscript

Response to Reviewer 2

We thank Reviewer 2 for the positive evaluation of the manuscript and for recognizing the public health relevance and practical implications of this work. No changes were requested.

We are grateful to the Academic Editor and reviewers for their insightful feedback. We believe these revisions have substantially strengthened the manuscript and improved its transparency, reproducibility, and alignment with PLOS ONE standards. We appreciate the opportunity to revise and resubmit and look forward to further consideration.

Sincerely,

Taylor Mullin

Taylor Mullin Ph.D., RN, CNOR

Mullin.94@osu.edu

614-404-3585

Attachment Submitted filename: PLOS One Review Letter.docx

# Editor report: Decision Letter 1

**DOI:** 10.1371/journal.pone.0349299.r003

**Authors:** Morufu Olalekan Raimi

29 Apr 2026

Trends in HPV Vaccination Initiation and Completion Among U.S. Adolescents Aged 13–17 Years, 2021–2023: Findings from the National Immunization Survey–Teen

PONE-D-26-11243R1

Dear Authors,

We’re pleased to inform you that your manuscript has been judged scientifically suitable for publication and will be formally accepted for publication once it meets all outstanding technical requirements.

Within one week, you’ll receive an e-mail detailing the required amendments. When these have been addressed, you’ll receive a formal acceptance letter and your manuscript will be scheduled for publication.

An invoice will be generated when your article is formally accepted. Please note, if your institution has a publishing partnership with PLOS and your article meets the relevant criteria, all or part of your publication costs will be covered. Please make sure your user information is up-to-date by logging into Editorial Manager at Editorial Manager® and clicking the ‘Update My Information' link at the top of the page. For questions related to billing, please contact billing support.

If your institution or institutions have a press office, please notify them about your upcoming paper to help maximize its impact. If they’ll be preparing press materials, please inform our press team as soon as possible -- no later than 48 hours after receiving the formal acceptance. Your manuscript will remain under strict press embargo until 2 pm Eastern Time on the date of publication. For more information, please contact onepress@plos.org.

Kind regards,

Morufu Olalekan Raimi, Ph.D

Academic Editor

PLOS One

Additional Editor Comments (optional):

Editorial Decision

Manuscript ID: PONE-D-26-11243_R1

Title: Trends in HPV Vaccination Initiation and Completion Among U.S. Adolescents Aged 13–17 Years, 2021–2023: Findings from the National Immunization Survey–Teen

Authors: Mullin et al.

Editor: Dr. Morufu Olalekan Raimi

Decision: Accept

I have completed the final review of your revised manuscript. The authors have responded thoroughly and point by point to all previous editorial and reviewer concerns, including those from Reviewer #1 regarding methodological transparency, reproducibility, and overstatement of “trends.” The current version now meets PLOS ONE’s standards for descriptive surveillance research.

Specifically, the authors have:

• Explicitly reframed the analysis as descriptive and stated that no formal hypothesis testing or trend testing was performed.

• Provided a step by step reproducible methodology, including exact variable names (HPVDOSE, HPVAGE1), ACIP 2 dose vs. 3 dose rules, handling of incomplete schedules, and the precise survey weight (PROVWT).

• Moved all key estimates with 95% confidence intervals into the main manuscript (Table 2), with a footnote clarifying the descriptive interpretation.

• Removed duplicated discussion text and tempered COVID 19 recovery language to a plausible hypothesis rather than a causal conclusion.

• Corrected reference formatting to Vancouver style, removed author biographies from the manuscript body, and corrected singular/plural declarations.

The remaining minor presentation issues (e.g., ensuring Figure 1 is high resolution, verifying all in text percentages have exact values) have been addressed adequately for publication.

No further revisions are required. The manuscript is methodologically sound, transparent, and provides a timely, policy relevant descriptive benchmark for HPV vaccination coverage during the post pandemic period.

I am pleased to accept the manuscript for publication in PLOS ONE.

Sincerely,

Dr. Morufu Olalekan Raimi

Academic Editor, PLOS ONE

Reviewers' comments:

# Editor report: Acceptance letter

**DOI:** 10.1371/journal.pone.0349299.r004

**Authors:** Morufu Olalekan Raimi

PONE-D-26-11243R1

PLOS One

Dear Dr. Mullin,

I'm pleased to inform you that your manuscript has been deemed suitable for publication in PLOS One. Congratulations! Your manuscript is now being handed over to our production team.

At this stage, our production department will prepare your paper for publication. This includes ensuring the following:

* All references, tables, and figures are properly cited

* All relevant supporting information is included in the manuscript submission,

* There are no issues that prevent the paper from being properly typeset

You will receive further instructions from the production team, including instructions on how to review your proof when it is ready. Please keep in mind that we are working through a large volume of accepted articles, so please give us a few days to review your paper and let you know the next and final steps.

Lastly, if your institution or institutions have a press office, please let them know about your upcoming paper now to help maximize its impact. If they'll be preparing press materials, please inform our press team within the next 48 hours. Your manuscript will remain under strict press embargo until 2 pm Eastern Time on the date of publication. For more information, please contact onepress@plos.org.

You will receive an invoice from PLOS for your publication fee after your manuscript has reached the completed accept phase. If you receive an email requesting payment before acceptance or for any other service, this may be a phishing scheme. Learn how to identify phishing emails and protect your accounts at https://explore.plos.org/phishing.

If we can help with anything else, please email us at customercare@plos.org.

Thank you for submitting your work to PLOS ONE and supporting open access.

Kind regards,

PLOS ONE Editorial Office Staff

on behalf of

Prof Morufu Olalekan Raimi

Academic Editor

PLOS One
